# N-Terminal 1–54 Amino Acid Sequence and Armadillo Repeat Domain Are Indispensable for P120-Catenin Isoform 1A in Regulating E-Cadherin

**DOI:** 10.1371/journal.pone.0037008

**Published:** 2012-05-16

**Authors:** Juanhan Yu, Yuan Miao, Hongtao Xu, Yang Liu, Guiyang Jiang, Maggie Stoecker, Endi Wang, Enhua Wang

**Affiliations:** 1 Department of Pathology, First Affiliated Hospital and College of Basic Medical Sciences, China Medical University, Shenyang, China; 2 Department of Pathology, Duke University Medical Center, Durham, North Carolina, United States of America; National Cancer Center, Japan

## Abstract

P120-catenin (p120ctn) exerts important roles in regulating E-cadherin and invasiveness in cancer cells. However, the mechanisms by which p120ctn isoforms 1 and 3 modulate E-cadherin expression are poorly understood. In the current study, HBE, H460, SPC and LTE cell lines were used to examine the effects of p120ctn isoforms 1A and 3A on E-cadherin expression and cell invasiveness. E-cadherin was localized on the cell membrane of HBE and H460 cells, while it was confined to the cytoplasm in SPC and LTE cells. Depletion of endogenous p120ctn resulted in reduced E-cadherin expression; however, p120ctn ablation showed opposite effects on invasiveness in the cell lines by decreasing invasiveness in SPC and LTE cells and increasing it in HBE and H460 cells. Restitution of 120ctn isoform 1A restored E-cadherin on the cell membrane and blocked cell invasiveness in H460 and HBE cells, while it restored cytoplasmic E-cadherin and enhanced cell invasiveness in SPC and LTE cells. P120ctn isoform 3A increased the invasiveness in all four cell lines despite the lack of effect on E-cadherin expression, suggesting a regulatory pathway independent of E-cadherin. Moreover, five p120ctn isoform 1A deletion mutants were constructed and expressed in H460 and SPC cells. The results showed that only the M4 mutant, which contains N-terminal 1–54 amino acids and the Armadillo repeat domain, was functional in regulating E-cadherin and cell invasiveness, as observed in p120ctn isoform 1A. In conclusion, the N-terminal 1–54 amino acid sequence and Armadillo repeat domain of p120ctn isoform 1A are indispensable for regulating E-cadherin protein. P120ctn isoform 1A exerts opposing effects on cell invasiveness, corresponding to the subcellular localization of E-cadherin.

## Introduction

To date, a number of regulatory mechanisms have been discovered involving carcinogenesis and tumor progression. Among these, increased experimental evidence has demonstrated that cadherin-mediated cell-cell interaction plays a pivotal role in the development and progression of many tumors [1], [2]. E-cadherin is a core component of epithelial cell-cell adhesion molecules, and its extracellular domain interacts in a homophilic, Ca^2+^-dependent fashion to form an adherens junction between neighboring cells. E-cadherin has been shown previously to participate in multiple aspects of cell processes, including development, morphogenesis and carcinogenesis [3], [4]. In many human cancers, reduced or abnormal expression of E-cadherin results in loss of cell-cell adhesion, which correlates with increased neoplastic cell proliferation, invasiveness and metastasis [5]–[8].

P120-catenin (p120ctn), a member of the catenin family, can interact directly with the intracellular domain of E-cadherin, and thus, plays important roles in regulating cell-cell adhesion [9]–[13]. Previous studies have demonstrated that p120ctn is essential for stabilization of E-cadherin molecules and for the anti-invasive properties of E-cadherin [10], [11], [14]. Loss, down-regulation, or delocalization of p120ctn results in loss of E-cadherin and correlates with the progression of several human tumors [10], [11], [15]–[17]. Recent studies, however, have suggested that p120ctn may have a function on tumor in two opposing directions by either promoting or suppressing tumor growth and invasiveness, depending on whether or not E-cadherin is expressed [18], [19].

P120ctn has four isoforms (isoforms 1 to 4) resulting from four transcription start sites [20] and additional isoforms are derived from three alternatively spliced exons A, B, and C [21], [22]. Although different isoforms have different N- or C-terminals, they share the central Armadillo repeat domain, which is essential for interacting with the juxtamembrane domain of E-cadherin on the cell membrane. While recent evidence has suggested that p120ctn isoforms regulate biological behavior of tumor cells by different mechanisms [22], [23], it is currently unclear how p120ctn isoforms 1 and 3 regulate E-cadherin and invasiveness in different tumor cells with distinct subcellular distribution of E-cadherin.

In the current study, we screened and eventually selected 1 human bronchial epithelial cell line (HBE) and 3 lung cancer cell lines (H460, SPC and LTE), of which E-cadherin is localized to the cell membrane in 2 and cytoplasm in the other 2 cell lines, respectively, and knocked down p120ctn using small interfering RNA (siRNA). P120ctn isoforms 1A or 3A were then restituted in the cells to investigate the effects on E-cadherin expression and cell invasiveness. In addition, multiple p120ctn isoform 1A deletion mutants were constructed and expressed in the p120ctn depleted cells to test which peptide domains are essential for the different function of p120ctn isoforms 1A and 3A.

## Materials and Methods

### Cell culture

Human bronchial epithelial cell line HBE and lung adenocarcinoma cell line SPC-A-1 were obtained from the American Type Culture Collection (Manassas, VA, USA). Human pulmonary giant cell carcinoma cell line NCI-H460 and human lung adenocarcinoma cell line LTEP-α-2 were obtained from the Cell Bank of Chinese Academy of Science (Shanghai, China). The cells were cultured in RPMI 1640 medium (Invitrogen, Carlsbad, CA, USA) containing 10% fetal calf serum (Invitrogen), 100 IU/ml penicillin (Sigma, St. Louis, MO, USA) and 100 µg/ml streptomycin (Sigma).

### Plasmid construction and transfection

**Figure 1:**
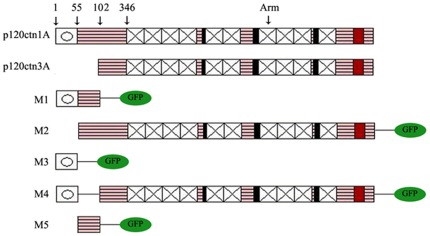
P120ctn isoform 1A, isoform 3A and five p120ctn 1A deletion mutants M1 to M5. P120 isoform 1A contains the coiled-coil domain and a central armadillo domain. P120 isoform 3A lacks the coiled-coil domain. Five p120ctn 1A deletion mutants M1 to M5 fused to green fluorescent protein (GFP): M1 contains only N-terminal 1–101 amino acids; M2 has N-terminal 1–54 amino acids deleted; M3 contains only N-terminal 1–54 amino acids; M4 has N-terminal 55–101 amino acids deleted; M5 contains only N-terminal 55–101 amino acids.

GFP-siRNA-P120ctn plasmids and p120ctn isoforms 1A and 3A cDNA plasmids have been described previously [24]. Five p120ctn 1A deletion mutants M1 to M5 fused to GFP were constructed by TaKaRa (TaKaRa, DaLian, China); M1: only N-terminal 1–101 amino acids; M2: deletion of N-terminal 1–54 amino acids; M3: only N-terminal 1–54 amino acids; M4: deletion of N-terminal 55–101 amino acids; M5: only N-terminal 55–101 amino acids (Figure 1).

The cells were transiently transfected with GFP-siRNA-p120ctn plasmids. 24 hours after transfection, aliquot cells were then transfected with cDNA plasmids of p120ctn isoform 1A, isoform 3A or each of the five p120ctn isoform 1A deletion mutants M1–M5. The transfection was performed with Lipofectamine 2000 (Invitrogen, Carlsbad, CA) or Attractene Transfection Reagent (QIAGEN GmbH, Hilden, Germany) according to the manufacturers' instruction.

### Western blot

Each protein sample (50 µg) was separated by SDS-PAGE. After transferring to a polyvinylidene fluoride (PVDF) membrane, the membrane was incubated overnight at 4°C with either the mouse monoclonal antibody against p120ctn (1∶500, 610134, BD Transduction Laboratories, USA), E-cadherin (1∶200, SC-8426, Santa Cruz Biotechnology, Santa Cruz, CA, USA) or GFP (1∶400, SC-9996, Santa Cruz Biotechnology, Santa Cruz, CA, USA). After incubating with peroxidase-coupled anti-mouse IgG (1∶2000, ZB-2305, Zhongshan Biotechnology, Beijing, China) at 37°C for 2 hours, the protein bands were visualized using ECL (Pierce, Rockford, IL, USA) and detected using the BioImaging Systems (UVP Inc.). The relative protein levels were calculated in reference to the amount of β-actin protein.

### Immunofluorescent staining

Cells grown on glass coverslips were fixed with ice-cold 4% paraformaldehyde for 30 minutes, followed by permeabilization with 0.2% Triton X-100. Primary antibodies are as follows: anti-p120ctn mouse monoclonal antibody (1∶200, 610134, BD Transduction Laboratories, USA), anti-E-cadherin rabbit polyclonal antibody (1∶100, SC-7870, Santa Cruz Biotechnology, CA, USA) and anti-E-cadherin mouse monoclonal antibody (1∶100, SC-8426, Santa Cruz Biotechnology, CA, USA). Primary antibodies were applied overnight at 4°C followed by incubation with secondary antibody conjugated to rhodamine/fluorescein isothiocyanate (FITC)-labeled goat anti-mouse or anti-rabbit antibodies (1∶100, ZF-0313 and ZF-0311, Zhongshan Biotechnology, Beijing, China). The nuclei were counterstained with propidium iodide/4, 6 diamidino-2-phenylindole. The cells were examined with an Olympus IX51 fluorescent microscope (Olympus, Tokyo, Japan), and images were captured with a CoolPIX 5400 camera (Nikon, Japan).

### Matrigel invasion assay

The assay was performed according to the manufacturer's instructions. In each upper chamber, 5×10^5^ cells were grown in serum-free medium on 8-µm porous polycarbonate membranes (Corning, Acton, MA, USA), which were coated with Matrigel basement membrane matrix (BD Biosciences, San Jose, CA, USA). The lower chambers were filled with RPMI 1640 medium containing 10% fetal calf serum. After incubation for 24 h at 37°C in a humid atmosphere with 5% CO2, the cells migrating through the pores were fixed with methanol for 30 min and stained with hematoxylin (Sigma). For each filter, the numbers of cells in five different fields of 400× magnification were counted visually using a Nikon E200 microscope. Each experiment was performed in triplicate.

### Statistical analysis

All statistical analyses were performed using SPSS for Windows software. Data from experimental groups were compared to those of control groups using the Independent-Samples T test. P values less than 0.05 were considered statistically significant.

## Results

### Depletion of endogenous p120ctn resulted in reduced E-cadherin but showed different effects on the cell invasiveness corresponding to the subcellular localization of E-cadherin

**Figure 2:**
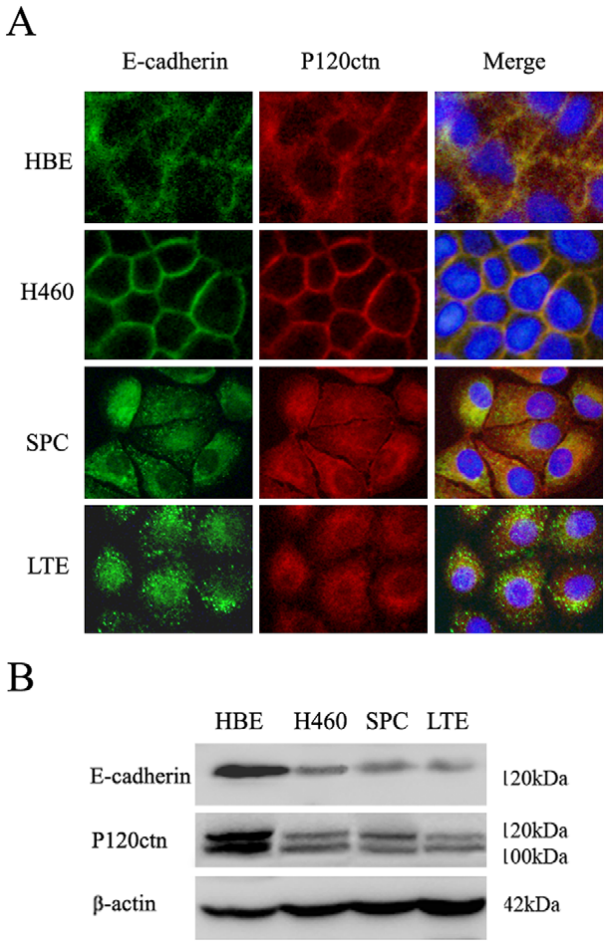
Expression and localization of p120ctn and E-cadherin. (**A**) By the immunofluorescence method, the expression of E-cadherin and p120ctn were noted to be restricted to the cell membrane at cell-cell adherens junctions in HBE and H460cells, whereas in SPC and LTE cells, E-cadherin and P120ctn were confined to the cytoplasm. (**B**) Western blot analyses confirmed E-cadherin and p120ctn were expressed in all four cell lines. Of all p120ctn isoforms, only two isoforms, p120ctn isoforms 1 (120 kDa) and 3 (100 kDa), were detected.

**Figure 3:**
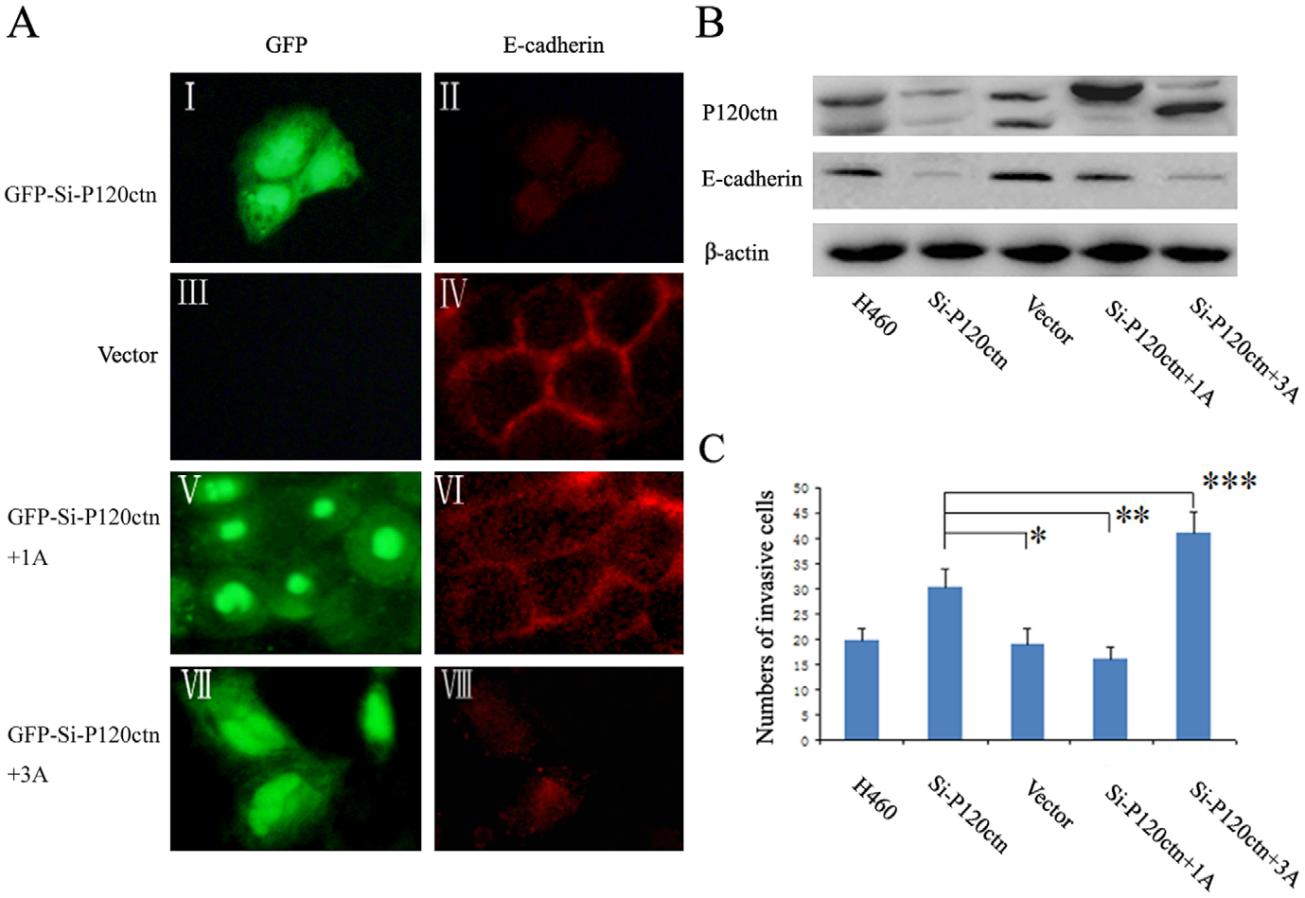
P120ctn isoform 1A restored E-cadherin on the cell membrane and suppressed the invasiveness in H460 cells. H460 cells were transiently transfected with GFP-siRNA-p120ctn plasmids or with empty vector as control. 24 hours after transfection, an aliquot of the cells was transfected again with p120ctn isoform 1A or 3A cDNA plasmids. (**A**) Levels and localization of E-cadherin were analyzed by immunofluorescence. The green signal shown in the nucleus and cytoplasm indicates effective expression of GFP from GFP-siRNA-P120ctn, confirming the successful transfection. Depletion of p120ctn (I) reduced the E-cadherin levels (II). Transfection with empty vector (III) did not affect the E-cadherin levels (IV). Restitution of p120ctn isoform 1A (V) restored the E-cadherin on the cell membrane (VI), while restitution of p120ctn isoform 3A (VII) had no effects on E-cadherin levels (VIII). (**B**) Levels of E-cadherin were then analyzed by Western blot assay. The results confirmed that depletion of p120ctn resulted in decreased E-cadherin levels; restitution of p120ctn isoform 1A restored the E-cadherin levels, while restitution of p120ctn isoform 3A had no effects on E-cadherin expression. (**C**) The invasiveness of H460 cells were analyzed by Matrigel invasion assay. P120ctn ablation enhanced the invasiveness in comparison with the control group transfected with vector alone (*, *P*<0.01). Restitution of p120ctn isoform 1A reduced the invasiveness of H460 cells in comparison with the group with p120ctn ablation (Si-p120ctn) (**, *P*<0.01), while p120ctn isoform 3A increased the invasiveness (***, *P*<0.01).

**Figure 4:**
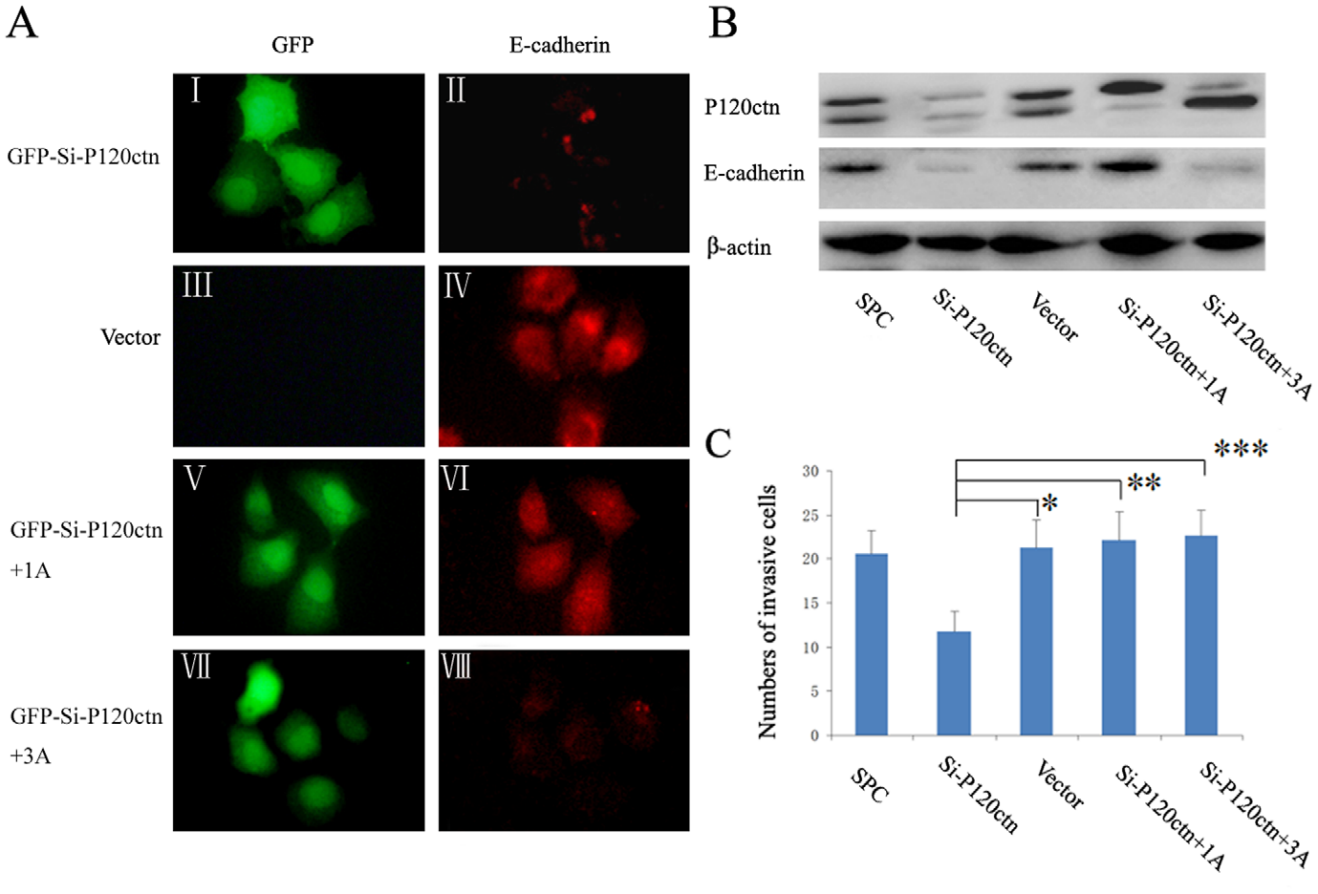
P120ctn isoform 1A restored the cytoplasmic E-cadherin levels and enhanced the invasiveness in SPC cells. SPC cells were transiently transfected with GFP-siRNA-p120ctn or with empty vector as control. 24 hours after transfection, an aliquot of cells was transfected again with p120ctn isoform 1A or 3A cDNA plasmids. (**A**) Levels and localization of E-cadherin were analyzed by immunofluorescence. The green signal shown in the nucleus and cytoplasm indicates effective expression of GFP from GFP-siRNA-P120ctn, confirming the successful transfection. Depletion of p120ctn (I) reduced the E-cadherin levels (II). Transfection with empty vector (III) did not affect the E-cadherin levels (IV). Restitution of p120ctn isoform 1A (V) restored the cytoplasmic E-cadherin levels (VI), while restitution of p120ctn isoform 3A (VII) had no effects on the E-cadherin levels (VIII). (**B**) Levels of E-cadherin were then analyzed by Western blot assay. The results confirmed that depletion of p120ctn resulted in decreased E-cadherin levels. Restitution of p120ctn isoform 1A restored the E-cadherin levels, while restitution of p120ctn isoform 3A had no effects on E-cadherin expression. (**C**) The invasiveness of SPC cells were analyzed by Matrigel invasion assay. P120ctn ablation reduced the cell invasiveness in comparison with the control group transfected with vector alone (*, *P*<0.01). Restitution of p120ctn isoform 1A and 3A both enhanced the invasiveness of SPC cells in comparison with the group with p120ctn ablation (Si-p120ctn) (**, *P*<0.01, ***, *P*<0.01).

The localization of E-cadherin and p120ctn in the cells was assessed using immunofluorescence. When cells were grown under conventional culture conditions, expression of E-cadherin and p120ctn were restricted to the cell membrane in HBE and H460 cells. On the other hand, in SPC and LTE cells, E-cadherin and p120ctn had similar distributions and were localized in the cytoplasm (Figure 2A). Western blot analysis detected mainly p120ctn isoforms 1 (120 kDa) and 3 (100 kDa) [20], [24], [25] in all four cell lines (Figure 2B). Ablation of endogenous p120ctn expression by siRNA resulted in decreased E-cadherin expression in all cell lines (Figure 3B and 4B, Figure S1B and S2B). Accordingly, immunofluorescence assays confirmed that E-cadherin was absent or undetectable on the membrane of H460 and HBE cells (Figure 3A and Figure S1A) and in the cytoplasm of SPC and LTE cells (Figure 4A and Figure S2A), respectively, after p120ctn ablation.

In addition, we examined the invasiveness of p120ctn knockdown cells by performing a serum-stimulated Matrigel invasion assay. P120ctn ablation enhanced the H460 and HBE cell invasiveness in comparison with the control (the group transfected with vector alone) (H460: 30.33±3.52 versus 18.93±3.22; *P*<0.01; HBE: 15.20±1.15 versus 9.33±1.40; *P*<0.01) (Figure 3C and Figure S1C); whereas in SPC and LTE cells, it reduced the cell invasiveness (SPC: 11.73±2.25 versus 21.27±3.17; *P*<0.01; LTE: 14.60±2.13 versus 20.73±2.58; *P*<0.01) (Figure 4C and Figure S2C).

### P120ctn isoform 1A and 3A showed different effects on E-cadherin expression and cell invasiveness

To explore the effects of p120ctn isoform 1A and isoform 3A on E-cadherin expression and cell invasiveness, all the cell lines were transiently transfected with cDNA plasmids of p120ctn isoform 1A or isoform 3A after depletion of endogenous p120ctn expression. Western-blot analysis demonstrated that E-cadherin protein levels were effectively restored by restitution of p120ctn isoform 1A in all the cell lines (Figure 3B and 4B, Figure S1B and S2B). Immunofluorescence assays confirmed that in H460 and HBE cells, restitution of p120ctn isoform 1A could restore E-cadherin on the cell membrane at cell-cell adherens junctions (Figure 3A and Figure S1A). On the other hand, E-cadherin remained in the cytoplasm in SPC and LTE cells after restitution of p120ctn isoform 1A (Figure 4A and Figure S2A). Interestingly, in comparison with the group with ablated p120ctn, restitution of p120ctn isoform 1A reduced the invasiveness of H460 and HBE cells (H460: 15.87±2.56 versus 30.33±3.52; *P*<0.01; HBE: 7.47±1.46 versus 15.20±1.15; *P*<0.01) (Figure 3C and Figure S1C), whereas in SPC and LTE cells, it increased the cell invasiveness (SPC: 22.13±3.27 versus 11.73±2.25; *P*<0.01; LTE: 23.07±3.15 versus 14.60±2.13; *P*<0.01) (Figure 4C and Figure S2C).

When p120ctn isoform 3A cDNA plasmid was transfected into HBE, H460, SPC or LTE cells depleted of p120ctn, the level of E-cadherin was not significantly changed (Figure 3A, B, Figure 4A, B, Figure S1A, B and Figure S2A, B). Despite this finding, in comparison with the group with ablated p120ctn, restitution of p120ctn isoform 3A demonstrated an increase in cell invasiveness in H460 (40.80±4.43 versus 30.33±3.52; *P*<0.01) (Figure 3C), HBE (21.07±2.40 versus 15.20±1.15; *P*<0.01) (Figure S1C), SPC (22.60±2.92 versus 11.73±2.25; *P*<0.01) (Figure 4C) and LTE cell lines (23.33±3.37 versus 14.60±2.13; *P*<0.01) (Figure S2C), respectively. The data from all four cell lines are summarized in Table S1.

### N-terminal 1–54 amino acid sequence and Armadillo repeat domain are essential for the function of p120cnt isoform 1A on up-regulating E-cadherin

**Figure 5:**
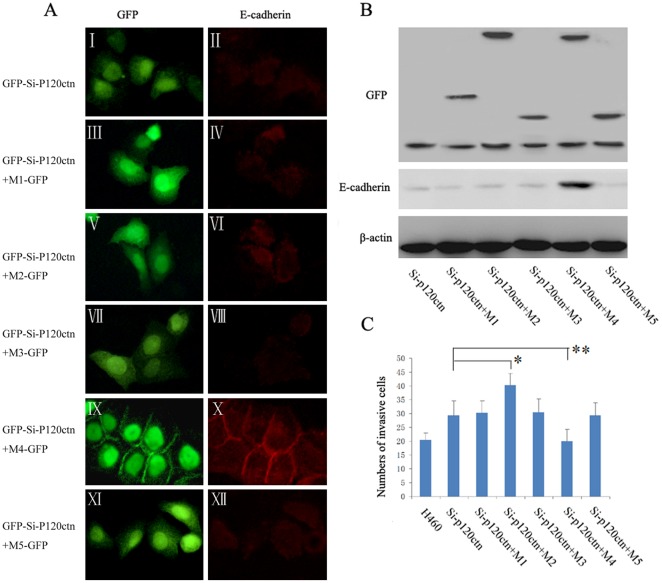
Mutant 4 restored the E-cadherin to cell membrane and suppressed cell invasiveness in H460 cells. H460 cells were transiently transfected with GFP-siRNA-p120ctn plasmids. 24 hours after transfection, an aliquot of the cells was transfected again with one of the five p120ctn isoform 1A deletion mutants M1–5 cDNA plasmids or with empty vector as control (The group transfected with vector alone was not included in the data). (**A**) Levels and localization of E-cadherin were analyzed by immunofluorescence. The green signal indicates expression of GFP from GFP-siRNA-P120ctn construct in image I and represents combined expression of GFP from GFP-siRNA-P120ctn and M1–5-GFP in images III, V, VII, IX and XI. GFP from GFP-si-P120ctn, M1–M3, and M5-GFP was expressed in the nucleus and cytoplasm. GFP from GFP-si-P120ctn and M4-GFP was expressed in the nucleus, cytoplasm and on the cell membrane (IX). Expression or repletion of M4 mutant (IX) restored E-cadherin on the cell membrane (X), while expression of the other mutants had no significant effects on E-cadherin levels. (**B**) The levels of E-cadherin were then analyzed by Western blot assay. Expression of M1–M5 mutants were detected by using antibody against GFP. The results showed that expression of M4 mutant up-regulated the E-cadherin levels, whereas the other mutants had no effects on the E-cadherin levels. (**C**) The invasiveness of H460 cells was analyzed by Matrigel invasion assay. Repletion of M4 mutant reduced the cell invasiveness in comparison with the group of p120ctn ablation (Si-p120ctn) (**, *P*<0.01), while repletion of M2 mutant enhanced the invasiveness (*, *P*<0.01). Repletion of the other mutants (M1, M3 and M5) did not show significant effects on cell invasiveness in comparison with the group of 120ctn ablation.

**Figure 6:**
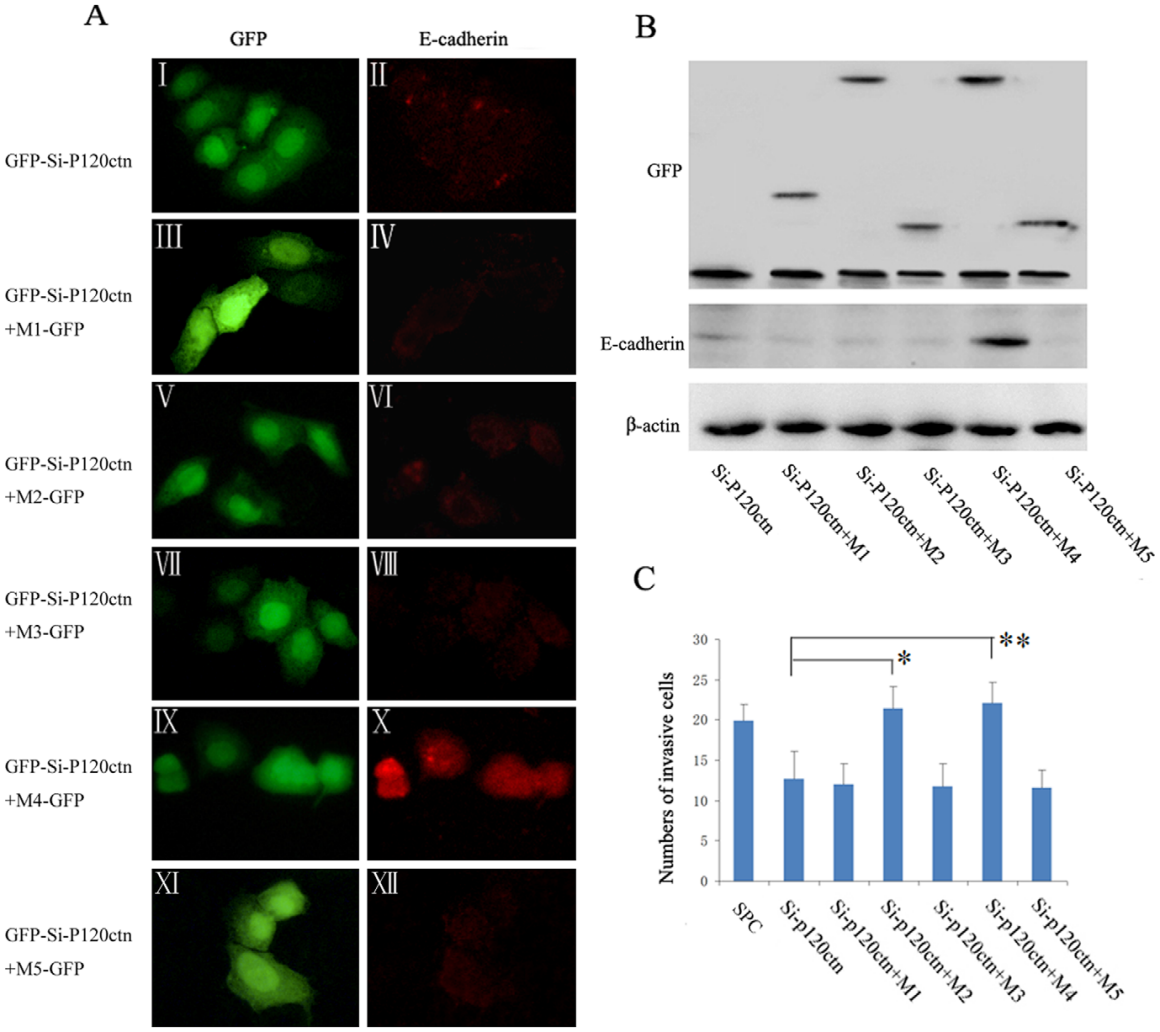
Mutant 4 restored cytoplasmic E-cadherin and enhanced the invasiveness in SPC cells. SPC cells were transiently transfected with GFP-siRNA-p120ctn plasmids. 24 hours after transfection, an aliquot of the cells was transfected again with one of the five p120ctn isoform 1A deletion mutants M1–5 cDNA plasmids or with empty vector as control (The group transfected with vector alone was not included in the data). (**A**) Levels and localization of E-cadherin were analyzed by immunofluorescence. The green signal indicates expression of GFP from GFP-siRNA-P120ctn construct in image I and represents combined expression of GFP from GFP-siRNA-P120ctn and M1–5-GFP in images III, V, VII, IX and XI. GFP from GFP-si-P120ctn or M1–M5-GFP was expressed in the nucleus and cytoplasm. Expression (repletion) of M4 mutant (IX) restored cytoplasmic E-cadherin (X), while expression of the other mutants had no significant effects on the E-cadherin levels. (**B**) The levels of E-cadherin were analyzed by Western blot assay. Expression of M1–M5 mutants was detected by using antibody against GFP. The results showed that expression of M4 mutant up-regulated E-cadherin levels, whereas expression of the other mutants had no effects on the E-cadherin levels. (**C**) The invasiveness of SPC cells was analyzed by Matrigel invasion assay. Expression of M2 or M4 mutants enhanced the cell invasiveness in comparison with the group with ablated p120ctn (Si-p120ctn) (*, *P*<0.01, **, *P*<0.01), while expression of other three p120 1A mutants did not show significant effects.

We have confirmed that p120ctn isoform 1A has effects on the regulation of E-cadherin expression, while p120ctn isoform 3A does not. Both p120ctn isoforms 1A and 3A contain the central Armadillo repeat domains; therefore, the variations of N-terminal structures may be responsible for the different functions of p120ctn isoform 1A and 3A. The N-terminal 1–54 amino acid sequence is unique to p120ctn isoform 1, which contains a coiled-coil domain (8–43 amino acids). The N-terminal 55–101 amino acids are present in p120ctn isoform 2A. Thereafter, five p120ctn isoform 1A deletion mutants M1 to M5 fused to GFP were constructed as described above (Figure 1) and transfected into p120ctn depleted H460 and SPC cells. Five p120ctn 1A deletion mutants were detected by Western-blot analysis using GFP antibody (Figure 5B, and 6B), and the results showed that expression of the M4 mutant, which contains N-terminal 1–54 amino acids and the Armadillo repeat domain, could restore the protein level of E-cadherin and corresponding changes of cell invasiveness in the two cell lines in the same fashion as seen in restitution of p120ctn isoform 1A (Figure 5 and 6). With regard to the localization of E-cadherin protein in the H460 and SPC cells, expression of M4 mutant resulted in a strong E-cadherin signal on the cell membrane at cell-cell adherens junctions in H460 cells depleted of p120ctn (Figure 5A), whereas in SPC cells depleted of p120ctn, E-cadherin signals were confined to the cytoplasm after repletion of M4 mutant (Figure 6A), as observed in restitution of p120ctn isoform 1A in the same cell line. Expression of M4 mutant reduced invasiveness of H460 cells in comparison with the group depleted of p120ctn as observed in restitution of p120ctn isoform 1A (20.07±4.27 versus 29.27±5.24; *P*<0.01) (Figure 5C), whereas in SPC cells, it increased invasiveness of cancer cells (22.13±2.53 versus 12.71±3.44; *P*<0.01) (Figure 6C).

In addition, M2 mutant was functionally the same as p120ctn isoform 3A: M2 mutant had no significant effect on E-cadherin expression but increased the invasiveness of both H460 (40.13±4.37 versus 29.27±5.24; *P*<0.01) (Figure 5C) and SPC cells (21.40±2.80 versus 12.71±3.44; *P*<0.01) (Figure 6C). The other mutants, M1, M3, and M5, had no effects on E-cadherin expression and invasiveness in H460 and SPC cells (Figure 5 and 6).

## Discussion

Consistent with previous studies [11], [15]–[17], we found that ablation of endogenous p120ctn resulted in noticeably decreased E-cadherin expression, to the extent that the immunofluorescence signal was hardly detected in all four cell lines adopted in this study, suggesting that p120ctn exerts an important role in E-cadherin stability [12]. The matrigel invasion assay demonstrated p120ctn ablation increased the invasiveness of HBE and H460 cells, whereas it reduced invasiveness of SPC and LTE cells. We and others have previously demonstrated that over-expression of p120ctn isoform 1A increases E-cadherin levels by a posttranscriptional mechanism [10], [24]. In this study, in addition to verifying that restitution of p120ctn isoform 1A could up-regulate the E-cadherin protein level by Western blot, we confirmed that the immunofluorescence signal of E-cadherin was restored on the cell membrane of HBE and H460 cells, but in SPC and LTE cells, E-cadherin remained in the cytoplasm after restitution of p120ctn 1A. Accordingly, restitution of p120ctn isoform 1A reversed the invasive phenotype induced by depletion of p120ctn in HBE and H460 cells, whereas, it restored the invasiveness of SPC and LTE cells. These data confirmed p120ctn isoform 1A indeed exerts opposing effects on cell invasiveness corresponding to the actual subcellular localization of E-cadherin. In HBE and H460 cells it is understandable that depletion of p120ctn might result in loss of E-cadherin-mediated cell-cell adhesion, leading to increased cell invasiveness. In accordance, restitution of p120ctn isoform 1A restored not only the level of E-cadherin, but also its localization to cell membrane, presumably leading to the subsequent recovery of E-cadherin-mediated cell-cell adhesion, and thus, decreased cell invasiveness. On the other hand, in the SPC and LTE cells in which E-cadherin is confined to the cytoplasm, restitution of p120ctn isoform 1A resulted in restored E-cadherin expression, but the E-cadherin remained in the cytoplasm, where it could not rescue cell-cell adhesion that is critical to restrict cell invasiveness. Our data are in agreement with the previous report that expression of p120 isoform 1A promotes invasiveness by regulating Rho GTPases activity in E-cadherin-deficient cancer cells [23].

It is interesting that restitution of p120ctn isoform 1A restored the E-cadherin levels and its localization on the cell membrane in HBE and H460 cells but did not alter cytoplasmic distribution of E-cadherin in SPC and LTE cells. These data suggest that p120ctn isoform 1A could up-regulate the E-cadherin protein levels, but p120ctn alone could not determine the destination of E-cadherin molecules. The actual localization of E-cadherin may be determined by a combination of factors including other catenin family members as well as non-catenin modulators.

Moreover, our data demonstrated that p120ctn isoform 1A contributes to the stabilization of E-cadherin in the cytoplasm. P120ctn may facilitate the trafficking of cadherins towards the cell membrane through its ability to interact with kinesin, and the co-immunoprecipitation data showed that p120ctn isoform 1 was much more strongly associated with kinesin than isoform 3 [26], [27]. In SPC and LTE cells, after restitution of p120ctn isoform 1A, newly synthesized E-cadherin might be initially delivered to the cell membrane but might be re-internalized shortly to the cytoplasm. Thereafter, by immunofluorescence staining, E-cadherin can be detected in the cytoplasm but not on the cell membrane in these two cell lines. While this quick turnover may prevent E-cadherin from degradation in these cells, its biological significance needs to be further studied.

In the current study, we demonstrated that p120ctn isoform 3A, though having no effects on E-cadherin protein levels, could enhance invasiveness in all four cell lines tested regardless of the expression status of E-cadherin. This effect on invasiveness in the cells independent of E-cadherin suggests a distinct pathway of p120ctn isoform 3A in regulating the progression of cancer cells. We and others have previously shown that Kaiso, which can recognize specific promoter sequences and inhibit the expression of target genes in the nucleus, interacts directly with p120ctn isoform 3A [28], [29]. Recent evidence has suggested that p120ctn, by binding to Kaiso, acts to relieve Kaiso-mediated transcriptional repression of certain genes [28], and in particular, our previous study has shown that restitution of p120ctn isoform 3A induced redistribution of Kaiso from the nucleus to cytoplasm of lung cancer cells [29]. These data, together with the findings in this study, suggest that up-regulation of p120ctn isoform 3A might hypothetically abrogate Kaiso-mediated repression of the target genes, such as MMP-7and MTA2, leading to increased invasiveness in the cells.

Both p120ctn isoform 1A and isoform 3A contain the central Armadillo repeat domain, which is essential for its interaction with the E-cadherin juxtamembrane domain. A previous study showed that there is no obvious difference in the interaction of p120ctn isoform 1A or 3A with E-cadherin on the cell membrane [10]. However, p120ctn isoform 1A and isoform 3A seemed to act differentially on modulating E-cadherin expression and cell invasiveness, according to our observation in this study. The structural variations of the two isoforms at the N-terminal of p120ctn might explain the difference in their biological functions. To address this issue, five p120ctn 1A N-terminal deletion mutants, M1 to M5, were constructed and transfected into the p120ctn-depleted cells, respectively. Western-blot analysis showed that only the M4 mutant could noticeably increase the protein level of E-cadherin both in H460 and SPC cells, while other mutants could not. A common feature for M1, M3 and M5 mutants is deletion of the Armadillo repeat domain, which is essential for p120ctn to bind E-cadherin, and the absence of this essential domain in these three mutants may explain their loss of function on E-cadherin expression. Additional experiments confirmed that the function of the M4 mutant was similar to that of p120ctn isoform 1A, and the M2 mutant, which has the N-terminal 1–54 amino acid sequences deleted, showed a function similar to that of p120ctn isoform 3A. These data highly suggest that the N-terminal 1–54 amino acid sequence and Armadillo repeat domain in p120ctn isoform 1A play a pivotal role in regulating E-cadherin level. This notion is supported by the fact that the coiled-coil sequence, which is localized to the N-terminal 1–54 amino acids in p120ctn, may participate in protein–protein interactions [30]. This N-terminal coiled-coil domain might stabilize E-cadherin directly or via a mechanism that includes its interaction with other protein factors. Future studies may focus on this domain using site-directed mutagenesis to alter the sequence of N-terminal 1–54 amino acids and by investigating the corresponding changes of protein function. These mutants with targeted alterations of the amino acid sequence with N-terminal 1–54 of CTNND1 could possibly elucidate the amino acids or regions essential for maintaining the conformation and/or the function of the coiled-coil domain in stabilizing E-cadherin. The N-terminal 55–101 amino acid sequence in isolation seemed to have no effect on E-cadherin expression based on the data in this study. The mechanisms by which p120ctn isoform 3A and its mutant counterpart M2 promote cell invasiveness are currently unclear and require further investigation.

It should be noted that the molecular weight of GFP is several times as big as the M1, M3 or M5 mutants with the Armadillo repeat domain deleted, and using it as a tag molecule to indicate transfection efficacy might compromise the intracellular localization of these p120ctn mutant isoforms. Nonetheless, the molecular weights of M2 and M4 mutants, both of which contain the armadillo domain, are over 100 kDa, much larger than GFP molecule. A distinct membranous fluorescence (M4-GFP) (Figure 5A, IX) and the restoration of membranous E-cadherin (Figure 5A, X) were seen on cells with co-transfection of GFP-Si-P120ctn and M4-GFP, whereas mutant 2, though containing armadillo repeat domain as well, did not demonstrate the effect, presumably due to deletion of N-terminal 1–54 amino acid sequence. Therefore, the expression and intracellular localization of p120ctn mutants as well as their effect on E-cadherin seem to depend upon certain intact domains rather than molecular weight of p120ctn mutants. Correlated expression of membranous p120ctn mutants and restoration of E-cadherin were not observed in co-transfection of other p120ctn mutant isoforms, probably owing to deletion of either armadillo repeat domain or N-terminal 1–54 amino acid sequence. Among 5 mutant isoforms, p120ctn M4 is the sole isoform containing intact Armadillo repeat domain and N-terminal 1–54 amino acids (Figure 1), and its effect on E-cadherin seems to be the same as that of p120ctn isoform 1A. Therefore, the findings highly suggest that the N-terminal 1–54 amino acid sequence and Armadillo repeat domain in p120ctn isoform 1A play a pivotal role in regulating E-cadherin protein expression and localization.

In conclusion, this study demonstrated that p120ctn isoform 1A up-regulates E-cadherin protein levels, while isoform 3A does not. Both isoforms showed an effect on cell invasiveness. While p120ctn isoform 1A has two opposing effects on cell invasiveness, corresponding to the distinct subcellular localization of E-cadherin in individual cell lines, p120ctn isoform 3A promotes cell invasiveness by an E-cadherin-independent mechanism. The N-terminal 1–54 amino acid sequence and Armadillo repeat domain in P120ctn isoform 1A are indispensable for regulating E-cadherin expression.

## Supporting Information

Figure S1 **Effects of p120ctn isoforms on E-cadherin expression and cell invasiveness in HBE cell line.** (A) Effect on E-cadherin expression by immunofluorescence staining. Note the absence to marked decrease in E-cadherin after knockdown of p120ctn by si-p120ctn transfection (II), in contrast to the membranous staining of E-cadherin in the cells transfected with vector alone (IV). Also note that restitution of p120ctn isoform 1A restores membranous expression of E-cadherin (VI), while restitution of isoform 3A shows only some cytoplasmic expression of E-cadherin with no significant membranous staining (VIII). Green signal indicates the expression of GFP from the reporter of the constructed plasmids, confirming an effective transfection. (B) Effect on E-cadherin expression by Western blot analysis. Note a markedly decreased E-cadherin level after si-p120ctn transfection and restoration of E-cadherin by restitution of p120ctn isoform 1A but not by isoform 3A. (C) Effect on cell invasiveness by Matrigel invasion assay. Note a marked increase in cell invasiveness after si-p120ctn transfection, corresponding to the knockdown of p120ctn (Figure S1B) and decrease in E-cadherin (Figure S1A and S1B). Restitution of p120ctn isoform 1A restores or suppresses the cell invasiveness to the original level, corresponding to the increase in E-cadherin seen in Figure S1A and S1B. Restitution of p120ctn isoform 3A shows enhanced cell invasiveness. These results are essentially the same as those obtained from the tests in H460 cells.
(TIF)

Figure S2 **Effects of p120ctn isoforms on E-cadherin expression and cell invasiveness in LTE cell line.** (A) Effect on E-cadherin expression by immunofluorescence staining. Note the absence to marked decrease in E-cadherin after knockdown of p120ctn by si-p120ctn transfection (II), in contrast to the cytoplasmic staining of E-cadherin without significant membranous expression in the cells transfected with vector alone (IV). Also note that restitution of p120ctn isoform 1A restores cytoplasmic expression of E-cadherin (VI), while restitution of isoform 3A shows only faint cytoplasmic staining of E-cadherin (VIII). Green signal indicates the expression of GFP from the reporter of the constructed plasmids, confirming an effective transfection. (B) Effect on E-cadherin expression by Western blot analysis. Note a significantly decreased E-cadherin level after si-p120ctn transfection and restoration of E-cadherin by restitution of p120ctn isoform 1A but no significant change by restitution of isoform 3A. (C) Effect on cell invasiveness by Matrigel invasion assay. Note a marked decrease in cell invasiveness after si-p120ctn transfection, corresponding to the knockdown of p120ctn (Figure S2B) and decrease in E-cadherin (Figure S2A and S2B). Restitution of p120ctn isoform 1A restores or enhances the cell invasiveness, corresponding to the increase in E-cadherin seen in Figure S2A and S2B. Interestingly, restitution of p120ctn isoform 3A shows enhanced cell invasiveness, despite its minimal effect on E-cadherin expression. These results are essentially the same as those obtained from the tests in SPC cells.
(TIF)

Table S1 **Effects of p120ctn isoforms on level/subcellular localization of E-cadherin and cell invasiveness in four cell lines.** While p120ctn isoform 1A up-regulates E-cadherin expression in all 4 cell lines tested, its effect on cell invasiveness seems to be in two opposing directions, corresponding to the subcellular distribution of E-cadherin in each cell line. In cells with endogenous membranous expression of E-cadherin (HBE and H460), up-regulation of E-cadherin by p120 isoform 1A seems to suppress cell invasiveness, whereas, in those with endogenous cytoplasmic expression (SPC and LTE), it appears to enhance cell invasiveness. P120ctn isoform 3A enhances cell invasiveness with no significant alteration of E-cadherin expression. Its effect on cell invasiveness seems to be independent of E-cadherin. *In comparison with the group transfected with vector alone. **In comparison with the group transfected with GFP-si-p120ctn.
(DOC)
