# Supplementary figures and images for: N-Terminal 1–54 Amino Acid Sequence and Armadillo Repeat Domain Are Indispensable for P120-Catenin Isoform 1A in Regulating E-Cadherin
Source: PLoS One. 2012 May 16;7(5):e37008. doi: 10.1371/journal.pone.0037008 (PMC3353978; doi:10.1371/journal.pone.0037008)

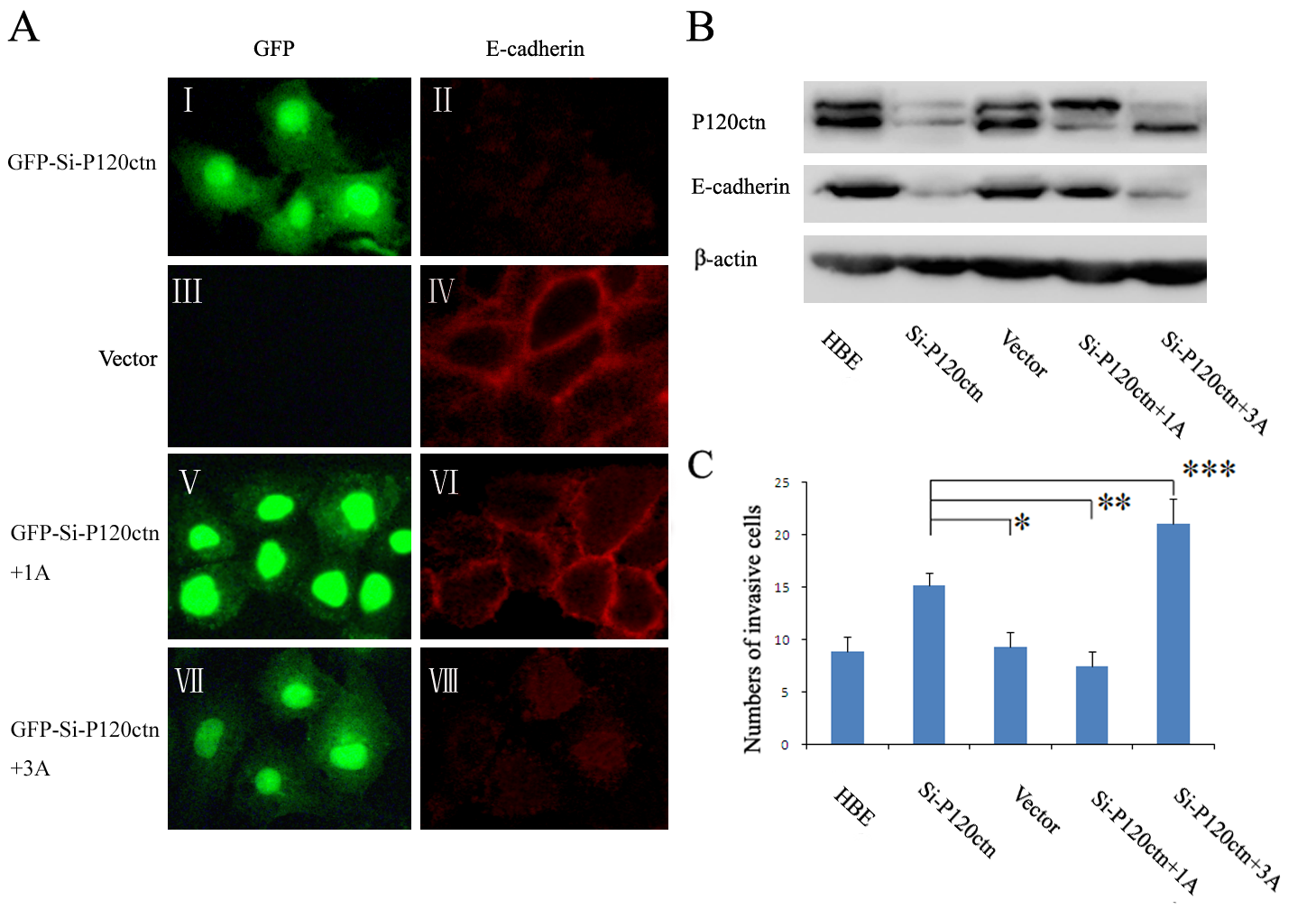

Supplement: Figure S1 — Effects of p120ctn isoforms on E-cadherin expression and cell invasiveness in HBE cell line. (A) Effect on E-cadherin expression by immunofluorescence staining. Note the absence to marked decrease in E-cadherin after knockdown of p120ctn by si-p120ctn transfection (II), in contrast to the membranous staining of E-cadherin in the cells transfected with vector alone (IV). Also note that restitution of p120ctn isoform 1A restores membranous expression of E-cadherin (VI), while restitution of isoform 3A shows only some cytoplasmic expression of E-cadherin with no significant membranous staining (VIII). Green signal indicates the expression of GFP from the reporter of the constructed plasmids, confirming an effective transfection. (B) Effect on E-cadherin expression by Western blot analysis. Note a markedly decreased E-cadherin level after si-p120ctn transfection and restoration of E-cadherin by restitution of p120ctn isoform 1A but not by isoform 3A. (C) Effect on cell invasiveness by Matrigel invasion assay. Note a marked increase in cell invasiveness after si-p120ctn transfection, corresponding to the knockdown of p120ctn (Figure S1B) and decrease in E-cadherin (Figure S1A and S1B). Restitution of p120ctn isoform 1A restores or suppresses the cell invasiveness to the original level, corresponding to the increase in E-cadherin seen in Figure S1A and S1B. Restitution of p120ctn isoform 3A shows enhanced cell invasiveness. These results are essentially the same as those obtained from the tests in H460 cells. (TIF) [file pone.0037008.s001.tif]

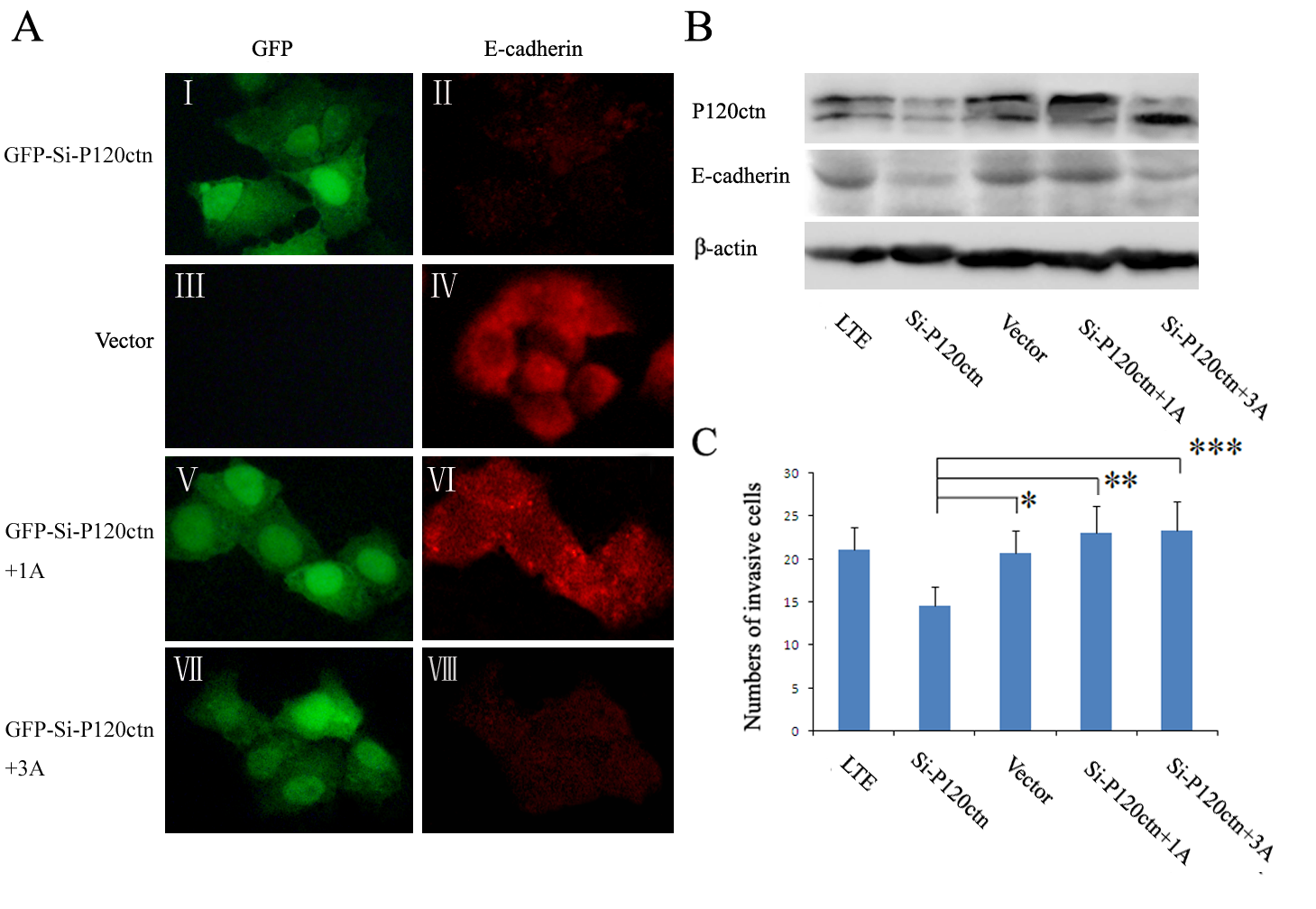

Supplement: Figure S2 — Effects of p120ctn isoforms on E-cadherin expression and cell invasiveness in LTE cell line. (A) Effect on E-cadherin expression by immunofluorescence staining. Note the absence to marked decrease in E-cadherin after knockdown of p120ctn by si-p120ctn transfection (II), in contrast to the cytoplasmic staining of E-cadherin without significant membranous expression in the cells transfected with vector alone (IV). Also note that restitution of p120ctn isoform 1A restores cytoplasmic expression of E-cadherin (VI), while restitution of isoform 3A shows only faint cytoplasmic staining of E-cadherin (VIII). Green signal indicates the expression of GFP from the reporter of the constructed plasmids, confirming an effective transfection. (B) Effect on E-cadherin expression by Western blot analysis. Note a significantly decreased E-cadherin level after si-p120ctn transfection and restoration of E-cadherin by restitution of p120ctn isoform 1A but no significant change by restitution of isoform 3A. (C) Effect on cell invasiveness by Matrigel invasion assay. Note a marked decrease in cell invasiveness after si-p120ctn transfection, corresponding to the knockdown of p120ctn (Figure S2B) and decrease in E-cadherin (Figure S2A and S2B). Restitution of p120ctn isoform 1A restores or enhances the cell invasiveness, corresponding to the increase in E-cadherin seen in Figure S2A and S2B. Interestingly, restitution of p120ctn isoform 3A shows enhanced cell invasiveness, despite its minimal effect on E-cadherin expression. These results are essentially the same as those obtained from the tests in SPC cells. (TIF) [file pone.0037008.s002.tif]
