# Supplementary material for: N-Terminal 1–54 Amino Acid Sequence and Armadillo Repeat Domain Are Indispensable for P120-Catenin Isoform 1A in Regulating E-Cadherin
Source: PLoS One. 2012 May 16;7(5):e37008. doi: 10.1371/journal.pone.0037008 (PMC3353978; doi:10.1371/journal.pone.0037008)
Supplement: Table S1 — Effects of p120ctn isoforms on level/subcellular localization of E-cadherin and cell invasiveness in four cell lines. While p120ctn isoform 1A up-regulates E-cadherin expression in all 4 cell lines tested, its effect on cell invasiveness seems to be in two opposing directions, corresponding to the subcellular distribution of E-cadherin in each cell line. In cells with endogenous membranous expression of E-cadherin (HBE and H460), up-regulation of E-cadherin by p120 isoform 1A seems to suppress cell invasiveness, whereas, in those with endogenous cytoplasmic expression (SPC and LTE), it appears to enhance cell invasiveness. P120ctn isoform 3A enhances cell invasiveness with no significant alteration of E-cadherin expression. Its effect on cell invasiveness seems to be independent of E-cadherin. *In comparison with the group transfected with vector alone. **In comparison with the group transfected with GFP-si-p120ctn. (DOC) [file pone.0037008.s003.doc]

| Cell line | Localization of E-cadherin and p120ctn | Si-p120ctn* | |  | Si-p120ctn+1A** | |  | Si-p120ctn+3A** | |
| --- | --- | --- | --- | --- | --- | --- | --- | --- | --- |
|  |  | Levels of E-cadherin | Cell invasiveness |  | Levels and localization of E-cadherin | Cell invasiveness |  | Levels and localization of E-cadherin | Cell invasiveness |
| HBE | Cell membrane | Decreased | Enhanced |  | Restored on the cell membrane | Reduced |  | Not changed | Enhanced |
| H460 | Cell membrane | Decreased | Enhanced |  | Restored on the cell membrane | Reduced |  | Not changed | Enhanced |
| SPC | Cytoplasm | Decreased | Reduced |  | Restored in the cytoplasm | Enhanced |  | Not changed | Enhanced |
| LTE | Cytoplasm | Decreased | Reduced |  | Restored in the cytoplasm | Enhanced |  | Not changed | Enhanced |
